# New Hydrophilic/Lipophilic Tetra-α-(4-carboxyphenoxy) Phthalocyanine Zinc-Mediated Photodynamic Therapy Inhibits the Proliferation of Human Hepatocellular Carcinoma Bel-7402 Cells by Triggering Apoptosis and Arresting Cell Cycle

**DOI:** 10.3390/molecules16021389

**Published:** 2011-02-07

**Authors:** Chunhui Xia, Yu Wang, Wei Chen, Wenxue Yu, Baiqi Wang, Tao Li

**Affiliations:** 1 Basic Medicine Department, Qiqihar Medical College, Qiqihar 161006, China; E-Mails: chunhuixia1969@sohu.com (C.X.); wyfr1970@sohu.com (Y.W.); 2 College of Materials Science and Engineering, Jilin University, Changchun 130012, China; E-Mail: slgwy@sohu.com (W.Y.); 3 College of Chemistry and chemical Engineering, Qiqihar University, Qiqihar 161006, China; E-Mail: chenwei150080@163.com (W.C.); 4 School of Public Health, Tianjin Medical University, Tianjin 300070, China; E-Mail: wbq1999@126.com (B.W.)

**Keywords:** tetra-α-(4-carboxyphenoxy) phthalocyanine zinc, photodynamic therapy, human hepatoma Bel-7402 cells, proliferation, apoptosis, cell cycle

## Abstract

Photodynamic therapy (PDT) is a novel and promising antitumor treatment. Phthalocyanine-mediated PDT has shown antitumor activity in some tumor cells, but the effect of new hydrophilic/lipophilic tetra-α-(4-carboxyphenoxy)phthalocyanine zinc (TαPcZn)-mediated PDT (TαPcZn-PDT) on human hepatocellular carcinoma Bel-7402 cells and underlying mechanisms have not been clarified. In the present study, therefore, the ultraviolet-visible (UV-vis) absorption spectrum and cellular localization of TαPcZn, and effect of TαPcZn-PDT on the proliferation, apoptosis, cell cycle, Bcl-2 and Fas in Bel-7402 cells were investigated by spectrophotometry, inverted microscope, 3-(4,5-dimethylthiazol-2-yl)-2,5-diphenyl-tetrazolium bromide (MTT) assay, electron microscopy, annexinV-FITC/propidium iodide double staining, DNA content and immunoblot assay, respectively. We found that an intense absorption in UV-vis absorption spectrum of TαPcZn was in the red visible region at 650–680 nm, where light penetration in tissue is efficient, that green TαPcZn localized to both plasma membrane and nuclear membrane of Bel-7402 cells, signifying that there was a selective uptake of TαPcZn in Bel-7402 cells and TαPcZn-PDT would be expected to directly damage DNA, and that TαPcZn-PDT significantly resulted in the proliferation inhibition, apoptosis induction, S cell cycle arrest, and down-regulation of Bcl-2 and Fas. Taken together, we conclude that TαPcZn-PDT inhibits the proliferation of Bel-7402 cells by triggering apoptosis and arresting the cell cycle.

## 1. Introduction

Hepatocellular carcinoma is one of the most common malignant tumors in the world. Currently, the traditional therapies of surgery, chemotherapy and radiation therapy play an important role in the systemic treatment of hepatocellular carcinoma. However, the treatment outcome is generally poor. Thus, it is very important to find an effective alternative treatment for hepatocellular carcinoma.

Photodynamic therapy (PDT) is a novel and promising antitumor treatment in which photosensitizing drugs that are excited with an appropriate wavelength of light significantly result in photochemical destruction of tumors by yielding reactive oxygen species (ROS), such as singlet oxygen and free radicals [1,2,3]. Compared with traditional therapies, the outstanding advantage of PDT is that it destroys tumor through selective uptake of photosensitisers and precise application of the light with modern fiber-optic systems, and does not harm the normal surrounding tissues seriously.

**Figure 1:**
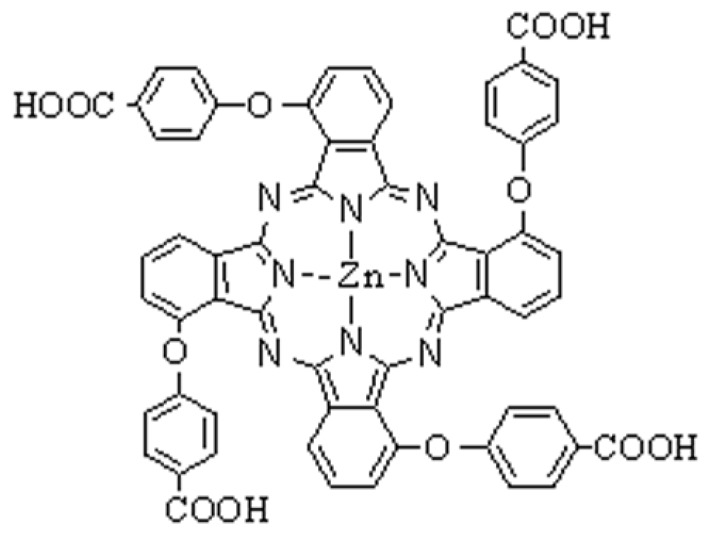
Chemical structure of TαPcZn.

Photosensitisers play a key role in PDT. At present, only a few porphyrin photosensitisers (Porfimer sodium, Talaporfin, Temoporfin, Verteporfin) are approved for the treatment of cancer in humans. Although these photosensitisers have demonstrated a wider spectrum of antitumor effects, they have various deficiencies that spur the development of better photosensitiser candidates [4,5]. As a result of the desirable electronic absorption and photophysical properties, phthalocyanines, containing a planar macrocycle with an 18 π-electron system, are one of the most potential photosensitiser candidates [6,7]. Furthermore, inasmuch as hydrophilic group redounds to the transport of drug in the body and lipophilic group conduces to the uptake of drug in cancer cells, phthalocyanines with hydrophilic/lipophilic structure may become promising candidates for selective photosensitisers. Recently, accumulated evidences have demonstrated highly selective growth inhibitory effects of hydrophilic/lipophilic phthalocyanines-mediated PDT in a variety of cancer cells [8,9,10]. However, the effect of new hydrophilic/lipophilic tetra-α-(4-carboxyphenoxy) phthalocyanine zinc (TαPcZn, Figure 1)-mediated PDT (TαPcZn-PDT) on human hepatocellular carcinoma Bel-7402 cells and underlying mechanisms have not been clarified.

Apoptosis or programmed cell death is a form of cell death in which a programmed sequence of events leads to the elimination of cells without releasing harmful substances into the surrounding area. Apoptosis plays a crucial role in developing and maintaining health by eliminating old cells, unnecessary cells, and unhealthy cells [11,12]. Nowadays, a number of studies have shown that apoptosis is one key pathways in PDT process [13,14].

The cell cycle is the series of events that take place in a cell resulting in its division and duplication. It consists of four distinct phases: G_1_ phase (pre-synthesis), S phase (synthesis), G_2_ phase (collectively known as interphase) and M phase (mitosis). The G0 phase is a quiescent period where cells have exited from the cell cycle and have stopped dividing. Activation of each phase relies on the proper progression and completion of the previous one. What with rapid and uncontrollable proliferation being one hallmark of cancer cell, arresting cancer cell cycle may offer therapeutic possibilities for treating malignant tumors. Accumulated evidence has demonstrated that cell cycle arrest leads to cell growth inhibition and/or apoptosis in PDT processes [15,16].

In the present study, the objective of our study is to investigate whether hydrophilic/ lipophilic TαPcZn-PDT inhibits the proliferation of Bel-7402 cells by triggering apoptosis and arresting cell cycle. Based on an *in vitro* model, we found that TαPcZn-PDT inhibited proliferation and induced apoptosis in Bel-7402 cells, simultaneously arresting the cells at S phase with concomitant down-regulation of Bcl-2 and Fas.

## 2. Results and Discussion

### 2.1. Ultraviolet-visible absorption spectrum of TαPcZn

**Figure 2:**
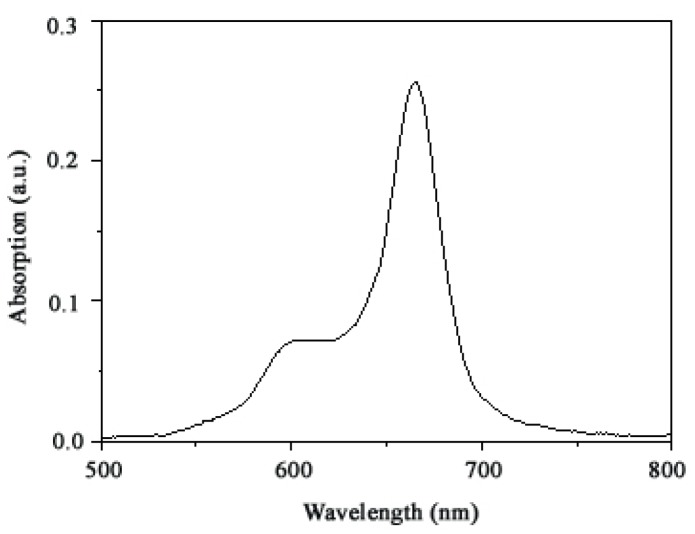
UV-vis absorption spectra of TαPcZn in DMSO/water (1:3, v/v) mixtures.

An ideal photosensitizer should have a good absorption of tissue-penetrating red light. The ultraviolet-visible (UV-vis) absorption spectrum of TαPcZn in dimethyl sulfoxide (DMSO)/water mixtures showed an intense absorption in the red visible region at 650–680 nm (Figure 2). It is clear that, compared with the strong absorption of porphyrin photosensitisers such as Porfimer sodium (λ_max absorption_ = 400 nm), Temoporfin (λ_max absorption_ =415 nm), and Talaporfin (λ_max absorption_ = 400 nm), TαPcZn exhibited an about 270 nm red-shifted absorption resulting in a better light penetration, higher PDT efficiency and lower skin phototoxicity [17,18]. Moreover, the electronic absorption spectra of TαPcZn can be readily interpreted using Gouterman’s four orbital model [19]. Specifically, the intense absorption is attributed to the transition from the highest occupied molecular orbital to the lowest unoccupied molecular orbital.

### 2.2. Inhibitory effect of TαPcZn-PDT on the proliferation of Bel-7402 cells and human dermal fibroblasts

**Figure 3:**
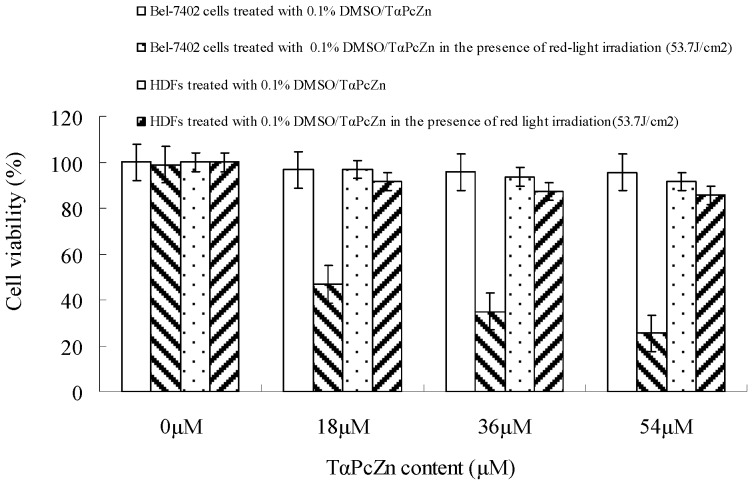
Effect of TαPcZn-PDT on the proliferation of Bel-7402 cells and HDFs. Proliferation of Bel-7402 cells and HDFs analyzed by MTT assay. Bel-7402 cells and HDFs were treated with the different concentrations of TαPcZn in the presence of red-light irradiation (53.7 J/cm^2^). Cell viability was detected by MTT assay. *P* < 0.01 *versus* the control value (cells treated with 0.1% DMSO).

From the biochemical aspect, an ideal photosensitizer should be non-toxic in the dark but after being exposed to light exhibit potent cytotoxic activity. Although a number of evidences have demonstrated that ideal phthalocyanine photosensitizers-mediated PDT can apparently inhibit proliferation in a variety of cancer cells [7,8,9,10,20,21,22,23,24], we firstly report the effect of hydrophilic/lipophilic TαPcZn-PDT on proliferation of Bel-7402 cells by the 3-(4,5-dimethylthiazol-2-yl)-2,5-diphenyl-tetrazolium bromide (MTT) assay. As shown in Figure 3, the red light (600-700 nm) without TαPcZn did not exhibit any toxicity against Bel-7402 cells, and TαPcZn alone had little effect on the proliferation of Bel-7402 cells.

However, TαPcZn-PDT remarkably suppressed the proliferation of Bel-7402 cells in a dose-dependent pattern, indicating that the combination of TαPcZn and red light irradiation can apparently inhibit the proliferation of Bel-7402 cells. The inhibition rate in Bel-7402 cells after TαPcZn-PDT treatment was significantly difference at P < 0.01 compared with the control cells. Furthermore, Figure 3 also showed that TαPcZn-PDT had little effect on human dermal fibroblasts (HDFs), indicating that TαPcZn might be an effective and safe photosensitizer. The TαPcZn-PDT processes can be explained as follows [25]: (1) When irradiated with red light, TαPcZn absorbs energy and converts to a triplet state (^3^TαPcZn^*^), a process known as intersystem crossing; (2) ^3^TαPcZn^*^ interacts with water and oxygen in its molecular oxygen (O_2_) or triplet state (^3^O_2_) to generate ROS (^1^O_2_, H_2_O_2_, OH and·O^−^_2_); (3) The generated ROS result in destruction of Bel-7402 cells. The processes described above can be expressed as follows:

(1)TαPcZn+hν→Intersystem crossing3TαPcZn*(2)3TαPcZn*+O2+H2O→H2O2+⋅OH+⋅O2−(3)3TαPcZn*+3O2→1O2(4)ROS(H2O2,⋅OH,⋅O2−,1O2)+Bel-7402 cells→Destruction of Bel-7402 cells

### 2.3. TαPcZn cellular localization

**Figure 4:**
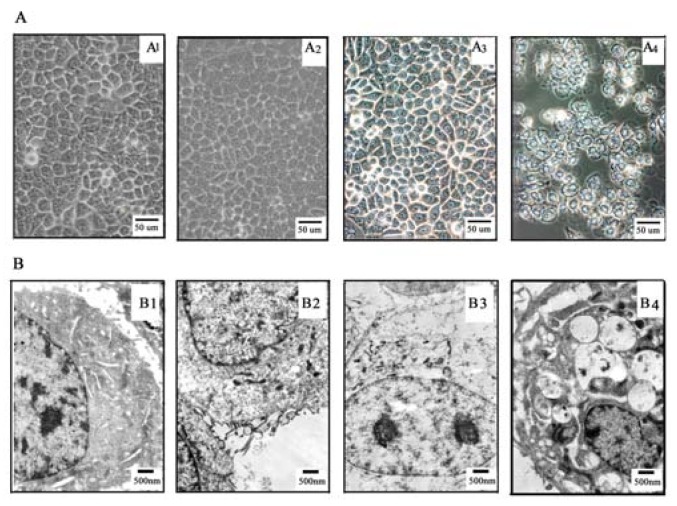
Effect of TαPcZn-PDT on Bel-7402 cells morphology. (A) Bel-7402 cells morphology analyzed by inverted microscope. Bars under each panel represent 50 um. (B) Bel-7402 cells morphology analyzed by Electron microscopy. Bars under each panel represent 500 nm. Morphology assay in: *panel A_1_ or B_1_*, control Bel-7402 cells treated with 0.1% DMSO; *panel A_2_ or B_2_*, Bel-7402 cells treated with red-light irradiation (53.7 J/cm^2^); *panel A_3_ or B_3_*, Bel-7402 cells treated with 54 μM TαPcZn; and *panel A_4_ or B_4_*, Bel-7402 cells treated with 54 μM TαPcZn in the presence of red-light irradiation (53.7 J/cm^2^).

To further assess inhibitory effect of TαPcZn-PDT, TαPcZn localization in Bel-7402 cells was detected by an inverted microscope. Previous studies suggested that phthalocyanine photosensitisers band to mitochondria, endoplasmic reticulum, and lysosomes [26,27]. The present inverted microscope assay showed that green TαPcZn selectively localized to both plasma membrane and nuclear membrane of Bel-7402 cells (Figure 4A_3_ and Figure 4A_4_), signifying that there was a selective uptake of TαPcZn in Bel-7402 cells and TαPcZn-PDT would be expected to directly damage DNA. In addition, it is clear that the hydrophilic carboxy/lipophilic phenyl structure led to the selective uptake of TαPcZn by Bel-7402 cells.

### 2.4. Effect of TαPcZn-PDT on the apoptosis of Bel-7402 cells

**Figure 5:**
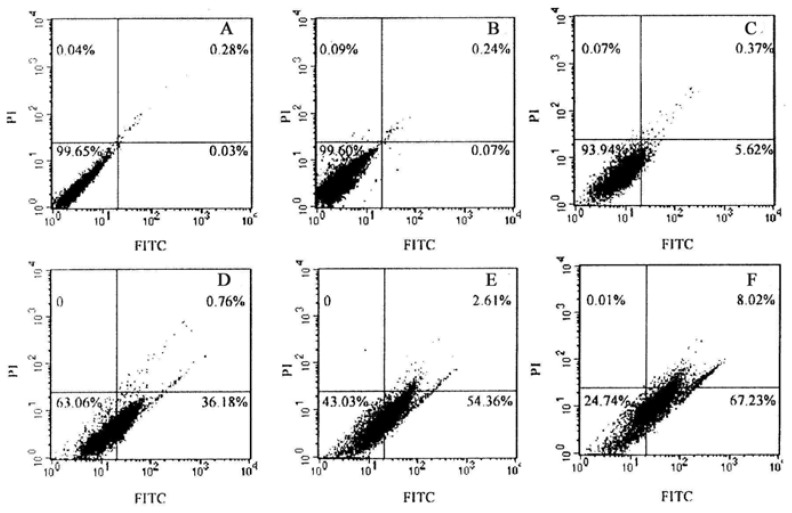
Effect of TαPcZn-PDT on the apoptosis of Bel-7402 cells assayed by flow cytometry analysis of Annexin V-FITC/PI double stained cells. Apoptosis assay in: *panel A*, control Bel-7402 cells treated with 0.1% DMSO; *panel B*, Bel-7402 cells treated with red-light irradiation (53.7 J/cm^2^); *panel C*, Bel-7402 cells treated with 54 μM TαPcZn; and *panel D~F*, Bel-7402 cells treated with 18, 36, 54 μM TαPcZn in the presence of red-light irradiation(53.7 J/cm^2^), respectively.

**Figure 6:**
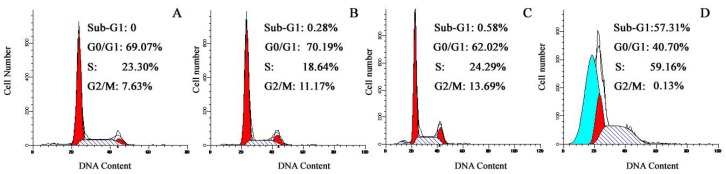
Effect of TαPcZn-PDT on the cycle and apoptosis of Bel-7402 cells assayed by flow cytometry analysis of DNA content. Assay of cycle and apoptosis in: *panel A*, control Bel-7402 cells treated with 0.1% DMSO; *panel B*, Bel-7402 cells treated with red-light irradiation (53.7 J/cm^2^); *panel C*, Bel-7402 cells treated with 54 μM TαPcZn; and *panel D*, Bel-7402 cells treated with 54 μM TαPcZn in the presence of red-light irradiation (53.7 J/cm^2^).

Accumulated evidences have suggested that phthalocyanines-mediated PDT can induce apoptosis in some cancer cells [22,23,24,25,28,29,30]. However, it is unclear whether TαPcZn-PDT can result in apoptosis of Bel-7402 cells. Therefore, in the present study, effect of TαPcZn-PDT on apoptosis of Bel-7402 cells was assayed by several ways. The morphological characteristics of apoptotic cell death were firstly detected by inverted microscope assay. Compared with control cells, light-treated cells and TαPcZn-treated cells, cells treated by TαPcZn-PDT apparently exhibited morphological characteristics of apoptotic cell death, such as cell shrinkage and extensive detachment of the cells from the cell culture substratum (Figure 4A_4_). The occurrence of apoptosis was further assessed by electron microcopy assay. The results also showed that TαPcZn-PDT obviously led to morphological characteristics of apoptotic cell death, such as cell shrinkage, nucleus and cytoplasm fragment, chromatin condensation, cytosolic vacuolation and apoptotic bodies formation (Figure 4B_4_). Furthermore, the apoptosis-eliciting effect of TαPcZn-PDT was quantitatively evaluated by flow cytometry analysis of annexin V-FITC/ propidium iodide (PI) double stained cells. Compared with the control treatment or light treatment alone, TαPcZn alone had little effect on the apoptosis of Bel-7402 cells (Figure 5). However, TαPcZn-PDT significantly led to the apoptosis of Bel-7402 cells in a dose-dependent pattern (Figure 5), suggesting that the combination of TαPcZn and red light can apparently induce the apoptosis of Bel-7402 cells. Similar results were obtained by assaying sub-G_1_ DNA content (Figure 6). All of the above results indicate that TαPcZn-PDT inhibits the proliferation of Bel-7402 cells by triggering apoptosis.

### 2.5. Effect of TαPcZn-PDT on the cycle of Bel-7402 cells

Accumulated results have demonstrated that phthalocyanines-mediated PDT can lead to cancer cell growth inhibition and/or apoptosis through G0/G1 or G2/M cell cycle arrest [22,23,24]. However, it is unclear whether TαPcZn-PDT can result in growth inhibition and/or apoptosis of Bel-7402 cell through arresting cell cycle. Therefore, in the present study, effect of TαPcZn-PDT on Bel-7402 cell cycle is investigated by DNA flow cytometry analysis. Compared with the control treatment, light treatment alone and TαPcZn treatment alone, TαPcZn-PDT apparently resulted in a decrease in the percentage of cells in G0/G1 and G2/M phase, and an increase in the percentage of cells in S phase (Figure 6), suggesting that TαPcZn-PDT arrested Bel-7402 cells at S phase and subsequently led to a decline in the percentage of cells in G2/M phase. Therefore, we conclude that TαPcZn-PDT inhibits the proliferation of Bel-7402 cell through S cell cycle arrest.

### 2.6. Effect of TαPcZn-PDT on Bcl-2 and Fas in TαPcZn-PDT-induced apoptosis of Bel-7402 cells

**Figure 7:**
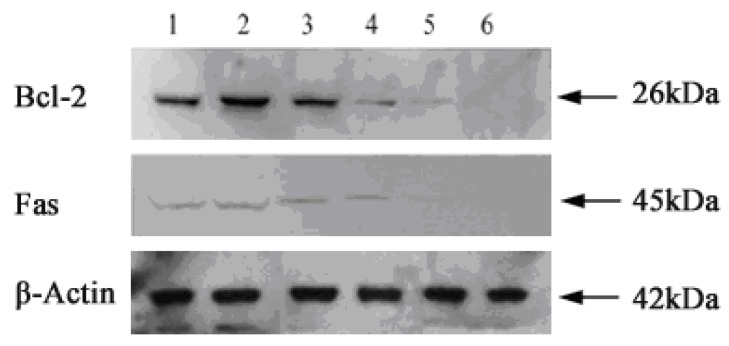
Effect of TαPcZn-PDT on Bcl-2 and Fas respectively in TαPcZn-PDT-induced apoptosis of Bel-7402 cells analyzed by Immunoblot assay. Expression of Bcl-2 and Fas in: *Lane 1*, control Bel-7402 cells treated with 0.1% DMSO; *Lane 2*, Bel-7402 cells treated with red-light irradiation (53.7 J/cm^2^); *Lane 3*, Bel-7402 cells treated with 54 μM TαPcZn; and *Lane 4~6*, Bel-7402 cells treated with 18, 36, 54 μM TαPcZn in the presence of red-light irradiation(53.7 J/cm^2^), respectively.

Bcl-2 is an anti-apoptotic protein. Fas is a 45-kD type II transmembrane receptor protein belonging to the tumor necrosis factor receptor family. Although Bcl-2 and Fas have been documented to play a crucial regulatory role in phthalocyanines-PDT-induced apoptosis [30,31,32], it is not clear whether Bcl-2 and Fas are involved in TαPcZn-PDT-induced apoptosis of Bel-7402 cells. Therefore, in the present study, effect of TαPcZn-PDT on Bcl-2 and Fas in TαPcZn-PDT-induced apoptosis of Bel-7402 cells is firstly investigated by Immunoblot assay. Compared with the control treatment, light treatment alone and TαPcZn treatment alone, TαPcZn-PDT dose-dependently led to down-regulation of Bcl-2 and Fas in TαPcZn-PDT-induced apoptosis of Bel-7402 cells (Figure 7), suggesting that Bcl-2 and Fas might modulate TαPcZn-PDT-induced apoptosis of Bel-7402 cells.

## 3. Experimental Section

### 3.1. Materials

Anti-Bcl-2 and anti-Fas antibodies were purchased from Santa Cruz Biotechnology (CA, USA). Annexin-V-FLUOS Staining Kit was purchased from Roche (Basel, Switzerland). Western blotting kit was purchased from Invitrogen (CA, USA). DMSO, MTT, PI, and RNAase were purchased from Sigma (St. Louis, MO, USA). RPMI-1640 medium was purchased from Gibco (CA, USA). Fetal bovine serum was purchased from PAA (Coelbe, Germany). Penicillin was purchased from Harbin Pharmaceutical Group (Heilongjiang, China). Streptomycin was purchased from Dalian Merro Pharmaceutical (Liaoning, China). Polyvinylidene difluoride membrane (PVDF) membrane was purchased from Amersham Pharmacia Biotech (Piscataway, NJ, USA). TαPcZn was synthesized as described in our previous report [33]. The TαPcZn stock solution was prepared in DMSO and stored at 4 °C in the dark. When used, the stock solution was appropriately diluted to obtain the desired concentration with a final DMSO concentration of 0.1%. All other chemicals and reagents were of analytic grade.

### 3.2. Ultraviolet-visible absorption spectrum assay

UV-vis absorption spectrum of TαPcZn in DMSO/water (1:3, v/v) mixtures was assayed by UV-2550 Spectrophotometer (Shimadzu, Kyoto, Japan).

### 3.3. Cell culture

Hepatocellular carcinoma Bel-7402 cells and HDFs were obtained from the Institute of Zoology, Chinese Academy of Science, China and cultured in RPMI-1640 medium supplemented with 10% fetal bovine serum and 100 U/mL penicillin-100 mg/L streptomycin in a humidified atmosphere of 5% CO_2_ at 37 °C.

### 3.4. TαPcZn-PDT treatment

Bel-7402 cells and HDFs in logarithmic growth-phase were seeded in culture plates and incubated for 24 h in a humidified atmosphere of 5% CO_2_ at 37 °C. After being rinsed with phosphate-buffered saline, the cells were treated with TαPcZn stock solution diluted in medium for 2.5 h at 37 °C in the dark. Then the cells were irradiated for 15 min with a SS-B instrument (Wuxi Holyglow Physiotherapy Instrument Co., Ltd., Jiangsu, China) emitting red light within the wavelength range of 600 to 700 nm. The light dose was 53.7 J/cm^2^ at an irradiance of 59.7 mW/cm^2^. Thereafter, cells were harvested at 8 h.

### 3.5. Cell viability assay

Cell viability was determined by MTT assay [34]. At 8 h post TαPcZn-PDT of Bel-7402 cells and HDFs, 20 µL of MTT working solution (5 mg/mL MTT in phosphate-buffered saline) was added in 96-well culture plates and incubated continuously at 37 °C for 4 h. All mediums were removed from wells and replaced with 150 µL of DMSO. After the blue-violet crystals were dissolved, the absorbance of each well was measured at 570 nm wavelength with a 680 microplate reader (Bio-Rad, CA, USA). The cell viability was calculated by the formula: cell viability = (absorbance of experimental wells/absorbance of control wells) × 100%. Values are means ±S.D. of three independent experiments.

### 3.6. Inverted microscope and electron microscopy assay

Following a 10.5-h incubation with 54 μM TαPcZn, TαPcZn localization in Bel-7402 cells was assayed by an IX70 inverted microscope (Olympus, Tokyo, Japan). Furthermore, Bel-7402 cells at 8 h post TαPcZn-PDT were also detected by an inverted microscope. After Bel-7402 cells at 8 h post TαPcZn-PDT were fixed with 3% glutaraldehyde in sodium cacodylate buffer (0.1M), they were transferred to phosphate buffer (0.1 M). The cells were then postfixed with 1% osmium tetroxide in S-collidine. After gradient dehydration in ethanol and acetone, the cells were transferred to propylene oxide, and embedded in Epon 812. Semi thin sections were stained with 1% methylene blue. Thereafter, the sample was sectioned into ultrathin slices, contrasted with uranyl acetate and lead citrate, and observed under a JEM1200EX transmission electron microscope (JEOL, Tokyo, Japan)

### 3.7. Flow cytometry analysis of Annexin V-FITC/PI double stained cells for apoptosis

Confirmation of apoptosis was determined by measurement of externalized phosphatidylserine residues as detected using annexin V-FITC. The harvested Bel-7402 cells were collected and washed with ice-cold phosphate-buffered saline, and then suspended in 500 µL of annexin V binding buffer A. 100 µL aliquot was taken, 2 µL of annexin V-FITC and 2 µL of PI were added, and the mixture was incubated for 5 min at room temperature in the dark. After the addition of 400 µL of binding buffer, 1 × 10^4^ cells were analyzed on a FACSCAN flow cytometer (Becton Dickinson, San Jose, CA, USA) by using cellquest software. The results are shown as a dotplot graph. In each graph, the percentages of apoptotic cells are indicated in lower right quadrant, the *Y*–axis corresponds to relative PI staining, and the *X*-axis corresponds to the log of the FITC signal.

### 3.8. DNA flow cytometry analysis for cell cycle and apoptosis

The harvested Bel-7402 cells were washed twice in phosphate-buffered saline, fixed with 70% ice-cold ethanol for 30 min, washed twice with phosphate-buffered saline, and stained with PI (50 μg/mL) containing RNAase (25 μg/mL) for 30 min. Cell cycle and apoptosis were assayed by DNA content on a flow cytometer.

### 3.9. Immunoblot assay

All immunoblots were performed using sodium dodecyl sulfate-polyacrylamide gel electrophoresis (SDS-PAGE) bis-Tris gel electrophoresis as outlined by the supplier. For total cellular protein, Bel-7402 cells were lysed in buffer containing 25 mM Hepes, pH 7.5, 0.3 M NaCl, 1.5 mM MgCl_2_, 0.2 mM EDTA, 0.1% Triton X-100, 20 mM β-glycerophosphate, 0.5 mM DTT, 1 mM sodium orthovanadate, 0.1 µM okadaic acid, and 1 mM phenylmethylsulfonyl fluoride. Protein concentrations of the cell lysates were determined by lorry method with bovine serum albumin as standard, and the supernatants were boiled in SDS sample buffer for 5 min. Equal amounts of lysate protein were run on 12% SDS–PAGE and electrophoretically transferred to PVDF membrane. After blocking, the blots were incubated with specific primary antibodies (anti-Bcl-2 and anti-Fas antibodies) overnight at 4 °C and further incubated for 1 h with horseradish peroxidase -conjugated respective secondary antibody. Bound antibodies were detected by enhanced chemiluminescence kit with a Lumino Image Analyzer (Founder, Beijing, China).

### 3.10. Statistical analysis

Values are means ±S.D. of three independent experiments. Statistical significance was determined using Student’s unpaired two-tailed *t*-test (SPSS 10.0 software). *P* value less than 0.01 was statistically significant.

## 4. Conclusions

In the present study, we found that an intense absorption in *UV-vis* absorption spectrum of TαPcZn was in the red visible region at 650–680 nm, that green TαPcZn localized to both plasma membrane and nuclear membrane of Bel-7402 cells, and that TαPcZn-PDT significantly resulted in the proliferation inhibition, apoptosis induction, S cell cycle arrest, and down-regulation of Bcl-2 and Fas. Taken together, we conclude that TαPcZn-PDT inhibits the proliferation of Bel-7402 cells by triggering apoptosis and arresting cell cycle.
